# Ocular pharmacological and biochemical profiles of 6-thioguanine: a drug repurposing study

**DOI:** 10.3389/fphar.2024.1375805

**Published:** 2024-03-25

**Authors:** Maria Consiglia Trotta, Carlo Gesualdo, Caterina Claudia Lepre, Marina Russo, Franca Ferraraccio, Iacopo Panarese, Ernesto Marano, Paolo Grieco, Francesco Petrillo, Anca Hermenean, Francesca Simonelli, Michele D’Amico, Claudio Bucolo, Francesca Lazzara, Filomena De Nigris, Settimio Rossi, Chiara Bianca Maria Platania

**Affiliations:** ^1^ Department of Experimental Medicine, University of Campania “Luigi Vanvitelli”, Naples, Italy; ^2^ Multidisciplinary Department of Medical, Surgical and Dental Sciences, University of Campania “Luigi Vanvitelli”, Naples, Italy; ^3^ Department of Mental, Physical Health and Preventive Medicine, University of Campania “Luigi Vanvitelli”, Naples, Italy; ^4^ Department of Pharmacy, University of Naples “Federico II”, Naples, Italy; ^5^ “Aurel Ardelean” Institute of Life Sciences, Vasile Goldis Western University of Arad, Arad, Romania; ^6^ Department of Biomedical and Biotechnological Sciences, School of Medicine, University of Catania, Catania, Italy; ^7^ Department of Precision Medicine, University of Campania “Luigi Vanvitelli”, Naples, Italy

**Keywords:** 6-thioguanine, diabetic retinopathy, melanocortin receptors, angiogenesis, drug discovery

## Abstract

**Introduction:**

The purine analog 6-thioguanine (6TG), an old drug approved in the 60s to treat acute myeloid leukemia (AML), was tested in the diabetic retinopathy (DR) experimental *in vivo* setting along with a molecular modeling approach.

**Methods:**

A computational analysis was performed to investigate the interaction of 6TG with MC1R and MC5R. This was confirmed in human umbilical vein endothelial cells (HUVECs) exposed to high glucose (25 mM) for 24 h. Cell viability in HUVECs exposed to high glucose and treated with 6TG (0.05–0.5–5 µM) was performed. To assess tube formation, HUVECs were treated for 24 h with 6TG 5 µM and AGRP (0.5–1–5 µM) or PG20N (0.5–1–5–10 µM), which are MC1R and MC5R antagonists, respectively. For the *in vivo* DR setting, diabetes was induced in C57BL/6J mice through a single streptozotocin (STZ) injection. After 2, 6, and 10 weeks, diabetic and control mice received 6TG intravitreally (0.5–1–2.5 mg/kg) alone or in combination with AGRP or PG20N. Fluorescein angiography (FA) was performed after 4 and 14 weeks after the onset of diabetes. After 14 weeks, mice were euthanized, and immunohistochemical analysis was performed to assess retinal levels of CD34, a marker of endothelial progenitor cell formation during neo-angiogenesis.

**Results:**

The computational analysis evidenced a more stable binding of 6TG binding at MC5R than MC1R. This was confirmed by the tube formation assay in HUVECs exposed to high glucose. Indeed, the anti-angiogenic activity of 6TG was eradicated by a higher dose of the MC5R antagonist PG20N (10 µM) compared to the MC1R antagonist AGRP (5 µM). The retinal anti-angiogenic effect of 6TG was evident also in diabetic mice, showing a reduction in retinal vascular alterations by FA analysis. This effect was not observed in diabetic mice receiving 6TG in combination with AGRP or PG20N. Accordingly, retinal CD34 staining was reduced in diabetic mice treated with 6TG. Conversely, it was not decreased in diabetic mice receiving 6TG combined with AGRP or PG20N.

**Conclusion:**

6TG evidenced a marked anti-angiogenic activity in HUVECs exposed to high glucose and in mice with DR. This seems to be mediated by MC1R and MC5R retinal receptors.

## 1 Introduction

Diabetic retinopathy (DR), one of the most severe diabetic complications, is the leading cause of blindness among working people in industrialized countries, with a significant socio-economic impact (Wild et al., 2004; Brownlee, 2005; Fletcher et al., 2005). The early and less severe DR form, known as non-proliferative DR (NPDR), is associated with long-term diabetes with inadequate glycemic control (Bloomgarden, 2009; Araszkiewicz and Zozulinska-Ziolkiewicz, 2016). NPDR is characterized by microaneurysms, microhemorrhages, retinal vascular abnormalities, exudates, and retinal occlusion (Park, 2016; Altmann and Schmidt, 2018). The progression to proliferative DR (PDR), the highly disabling pathology with the risk of pre-retinal hemorrhages and secondary retinal detachments (Dao-Yi et al., 2001), is triggered by the formation of the retinal ischemic area and the consequent stimulation of the vascular endothelial growth factor (VEGF) actions (Kollias and Ulbig, 2010). These lead to retinal neo-angiogenesis and increased vascular permeability (Arrigo et al., 2022).

Currently, anti-VEGF therapy is the pharmacological gold standard for DR; however, its management remains still challenging, with the 40% of inadequately treated NPDR cases evolving to PDR within 12 months (Hendrick et al., 2015; Wang and Lo, 2018). Therefore, new anti-angiogenic molecules targeting retinal vessel remodeling and angiogenesis could be considered novel pharmacological tools to prevent the DR progression (Lieth et al., 2000; Barber, 2003; Barber et al., 2011; Fehér et al., 2018). With regard to this finding, the purine analog 6-thioguanine (6TG), an old drug approved in the 60s to treat acute myeloid leukemia (AML), has shown a remarkable anti-angiogenic activity in AML experimental and clinical settings, by modulating endothelial cell motility, sprout formation, collagen gel invasion, and morphogenesis (Presta et al., 2002). Thus, 6TG could be repurposed as a drug to deal with pathologies characterized by pathological angiogenesis, such as DR.

We hereby identified, through a virtual screening approach, 6TG as a putative melanocortin receptor type 1 and type 5 (MC1R and MC5R) ligand, using the same *in silico* repurposing campaign, which was previously carried out by our group (Gesualdo et al., 2021). In this regard, since it has already been demonstrated that retinal MC1R and MC5R ligands exert an anti-angiogenic effect, beneficial for DR resolution (Maisto et al., 2017; Rossi et al., 2021), we have analyzed the binding of 6TG to MC1R and MC5R, through a structure-based computational approach. To confirm the computational findings, we investigated the interaction between 6TG and melanocortin receptors by using selective MC1R and MC5R antagonists in human umbilical vein endothelial cells (HUVECs) exposed to high glucose and in a mouse model of early DR.

## 2 Materials and methods

### 2.1 Molecular modeling, virtual screening, MM-GBSA calculations, and molecular dynamics simulations

Computational studies have been carried out within the Schrödinger Maestro environment, specifically using the modules of the drug discovery bundle. For methodology regarding homology modeling, molecular dynamics simulation, molecular docking, and virtual screening of approved FDA compounds, please refer to our previous paper authored by Gesualdo et al., 2021. Human MC1 and MC5 receptor (hMC1R and hMC5R) structures were modeled using the FASTA files from accession numbers Q01726.2 and NP_005904.1 as primary sequences for hMC1R and hMC5R, respectively. Homology models were built using the X-ray structure of human melanocortin receptor 4 (PDB:6W25) as a common template. Models were optimized through the all-atom molecular dynamics (MD) simulation of membrane protein systems in explicit water. MD trajectories were clustered, and clusters were chosen on the basis of affinity for known selective MC1R and MC5R compounds (Gesualdo et al., 2021). The molecular docking of 6TG was carried out with the extreme precision option of Glide within the Schrödinger Maestro environment. The complexes 6TG/hMC1R and 6TG/hMC5R were subjected to MM-GBSA calculations, and residues within 15 Å from the ligands were set free to move during the minimization protocol, applying the VSGB 2.0 implicit solvation model and implicit membrane model.

Therefore, 6TG/hMC1R and 6TG/hMC5R complexes were inserted in a 30 Å^3^ 1-palmitoyl-2-oleoyl-glycero-3-phosphocholine (POPC) lipid membrane system according to the output from the OMP database (https://opm.phar.umich.edu/). The TIP3P water model was selected, each system was neutralized, and NaCl was added to a final 150 mM concentration. The system ionization state was set for a pH = 7.4. Following the membrane protein equilibration protocol, 50 ns NPγT ensemble production runs were carried out. The simulations were analyzed, within the Schrödinger Maestro environment, and ligand–receptor interactions were described in terms of protein–ligand contact frequency, protein and ligand root mean deviation (RMSD), and protein–ligand root mean square fluctuation.

### 2.2 Compounds

6TG and streptozotocin (STZ) were purchased, respectively, from R&D system (Milan, Italy, 4061) and Santa Cruz Biotechnology (Heidelberg, Germany, sc-200719). The MC1R antagonist AGRP and the MC5R antagonist PG20N were synthetized as previously described (Carotenuto et al., 2015; Merlino et al., 2018; 2019).

### 2.3 Cell viability and tube formation assay

The effects of 6TG were assessed *in vitro* on HUVECs, purchased from Lonza (Milan, Italy), grown in a basal medium (cod. EGM2, Lonza), and enriched with SingleQuots™ (Palinski et al., 2021).

The 3-(4,5-dimethylthiazol-2-yl)-2,5-diphenyltetrazolium bromide (MTT) assay was carried out to determine cell viability, starting from 1 × 10^4^ HUVECs/well, seeded in 96-well plates (Li et al., 2017). HUVECs were then cultured for 14 h under normal (5 mmol/L) or high (25 mmol/L) glucose conditions (respectively, NG or HG groups) (Xu et al., 2020) and exposed for 24 h to 6TG dissolved in EGM-2 media (6TG groups) at different concentrations (0.05–0.5–5 µM). All the treatments were carried out in quadruplicate. At the end of the treatments, MTT (1:10, Elabscience) was added to the medium, and the plates were incubated at 37°C for 4 h. Then, the medium was removed, and dimethyl sulfoxide (DMSO, 150 μL/well) was added to solubilize the formazan crystals. Optical density (OD) at 570 nm was determined using a microplate reader (TECAN 2000 Infinity). HUVEC viability was expressed as a percentage (%) of the control group (NG).

For the tube formation assay, 6 × 10^4^ HUVECs were grown in μ-slide IbiDI culture plates with reduced Matrigel (GIBCO Lonza) (Palinski et al., 2021). NG or HG cells were exposed at 6TG 5 µM alone or in combination with MCR antagonists, dissolved in a phosphate saline buffer (PBS). Specifically, HUVECs were exposed to the following combinations: 6TG (5 µM) + AGRP (0.5–1–5 µM) and 6TG (5 µM) + PG20N (0.5–1–5–10 µM). All the treatments were carried out in quadruplicate. After capturing images using the ZEISS confocal microscope, the branch number for each field (*n* = 3) was quantified using ZEN Microscopy software (ZEISS, Germany).

### 2.4 Animals

For the induction of the *in vivo* DR model, a single dose of STZ (65 mg/kg) (Gesualdo et al., 2021) was used to induce diabetes in 6-week-old C57BL/6J male mice (23.4 ± 2.1 g) (Envigo, Italy). All the animal experimental procedures, in line with the Association for Research in Vision and Ophthalmology (ARVO) Statement for the Use of Animals in Ophthalmic and Vision Research, were approved by the Italian Ministry of Health (number 522/2019-PR, 19 July 2019) and by the Institutional Ethical Committee of the “Vasile Goldis” Western University of Arad (number 135, 1 March 2019). Mice had free access to standard chow and mineral water in single standard cages and were exposed to controlled temperature, humidity, and light/dark (12 h/12 h) cycle.

After an overnight fast, mice received a single intraperitoneal (i.p.) injection of sodium citrate (SCT) buffer (pH 4.5) as non-diabetic controls (CTR group) or STZ (65 mg/kg) freshly dissolved in 50 mM SCT (STZ group). Blood glucose levels were measured after 4 h fasting, using a one-touch glucometer (Accu-Chek Active, Roche Diagnostics, United States). Only STZ mice with blood glucose levels beyond 2.5 g/L on two consecutive weeks were considered diabetic (Gesualdo et al., 2021) and were included in the study to test the effects of intravitreal injections of 6TG. Because this study has been designed as a proof-of-concept, the intravitreal administration of 6TG was used to reach reproducible drug levels in the retina.

Four mice per group were randomized as follows: I—non-diabetic mice, used as controls (CTR group); II—non-diabetic mice receiving 6TG (2.5 mg/kg) intravitreally (CTR + 6TG); III—diabetic mice receiving PBS (pH 7.4) intravitreally (STZ group); IV–V–VI—diabetic mice receiving intravitreal injections of 6TG (0.5–1–2.5 mg/kg) (STZ + 6TG); VII–VIII—diabetic mice receiving intravitreal injections of the MC1R antagonist AGRP and 6TG (1–2.5 mg/kg) (STZ + 6TG + AGRP group); IX–X—diabetic mice receiving intravitreal injections of the MC5R antagonist PG20N and 6TG (1–2.5 mg/kg) (STZ + 6TG + PG20N).

**FIGURE 1:**
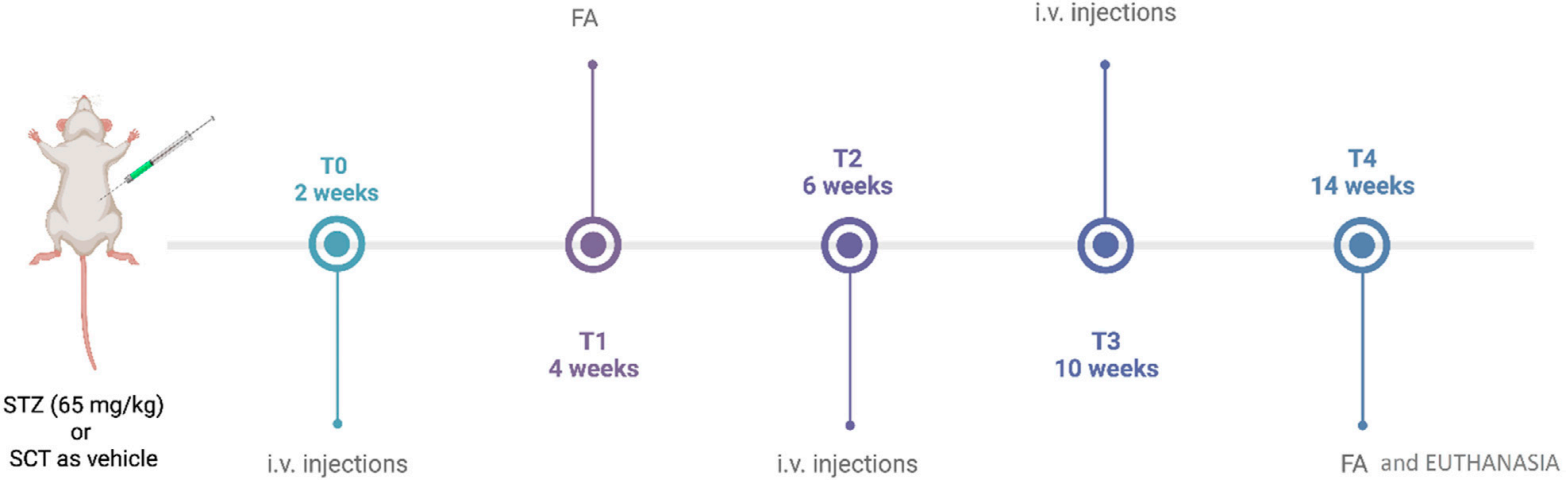
Timeline of the experimental design. STZ, streptozotocin; SCT, sodium citrate; FA, fluorescein angiography; i.v., intravitreal. This figure was created with BioRender.com.

Specifically, STZ and STZ + 6TG mice received intravitreal injections (5 µL) of PBS or 6TG (0.5–1–2.5 mg/kg) (Oancea et al., 2017) after 2 weeks from STZ injection (T0), and then every 4 weeks (at 4 and 8 weeks, respectively, indicated as T2 and T3) (Figure 1) (Gesualdo et al., 2021). 6TG was dissolved in DMSO and then diluted in PBS to a final concentration of 0.1% DMSO (Tsai et al., 2009; Seo and Suh, 2017). STZ + 6TG + ANTA MCR groups received intravitreal injections (5 µL) of the MC1R antagonist AGRP (14.3 µM in sterile PBS) or the MC5R antagonist PG20N (130 nM in sterile PBS) (Rossi et al., 2021) at T0, followed by intravitreal injections of 6TG (1–2.5 mg/kg) after 24 h. The same procedures were repeated every 4 weeks, at T2 and T3 (Figure 1).

### 2.5 Intravitreal injections and fluorescein angiography

For intravitreal injections, mice were anesthetized with pentobarbital (45 mg/kg in saline) and received tetracaine (1%) for local anesthesia into the right eye, along with tropicamide (5%) for pupils’ dilatation. PBS, 6TG, AGRP, and PG20N preparations (5 μL) were injected into the mice vitreous with a sterile syringe fitted with a 30-gauge needle (Micro-fine; Becton Dickinson AG, Meylan, France), after performing anterior chamber paracentesis (5 μL) to avoid an intraocular pressure increase (Biswas et al., 2007).

For fluorescein angiography (FA), mice received an i.p. injection of 10% fluorescein (1 mL/kg, AK-Fluor; Akorn, Inc.). Retinal vasculature was evaluated with a Topcon TRC-50DX apparatus (Topcon, Tokyo, Japan) in the same animal after 4 weeks from STZ (T1) and then after 12 weeks from T0 (T4) (Figure 1), the first time point reported to be associated with marked vessel alterations in the same DR mouse model (Butler and Sullivan, 2015; Gesualdo et al., 2021; Rossi et al., 2021). Vessel abnormalities (VAs), reported as mean observed at T1 and T4 by two different ophthalmologists unaware of the treatment, were scored at T4 as 0 = absence of VA; 1 = presence of vessel thinning; 2 = presence of vessel thinning and tortuosity; 3 = presence of thinning, tortuosity, and/or crushing; 4 = presence of vessel thinning and tortuosity, venous beading, and rosary-like vessels (Gesualdo et al., 2021).

After FA at T4, mice were euthanized to remove the eyes for immunohistochemical analysis. These were placed in cooled PBS, fixed in 10% neutral buffered formalin, and paraffin-embedded.

### 2.6 Immunohistochemistry

After deparaffinization, 5-μm ocular sections were incubated overnight at 4°C with the CD34 primary antibody (1:100; sc-74499 Santa Cruz Biotechnology, United States), which was used as a marker of endothelial progenitor cells (EPCs) during neo-angiogenesis (Di Filippo et al., 2014). After being washed with PBS, the sections were incubated with the biotin-conjugated anti-mouse IgG secondary antibody and avidin–biotin peroxidase complex (DBA, Milan, Italy). Then, six microscopic fields for each retina (*n* = 4 per group) were visualized at ×200 magnification and analyzed by an expert pathologist (intra-observer variability 5%) unaware of the experimental protocol. CD34 positive particles per area were expressed as % of the positive stained area/total area.

### 2.7 Statistical analysis

Statistical analysis was performed using GraphPad Prism v.8 (GraphPad Software, La Jolla, CA, United States). Differences were considered statistically significant for *p* values < 0.05 by one-way ANOVA, followed by Tukey’s multiple comparisons test.

## 3 Results

### 3.1 Virtual screening, molecular docking, and MM-GBSA calculations

**FIGURE 2:**
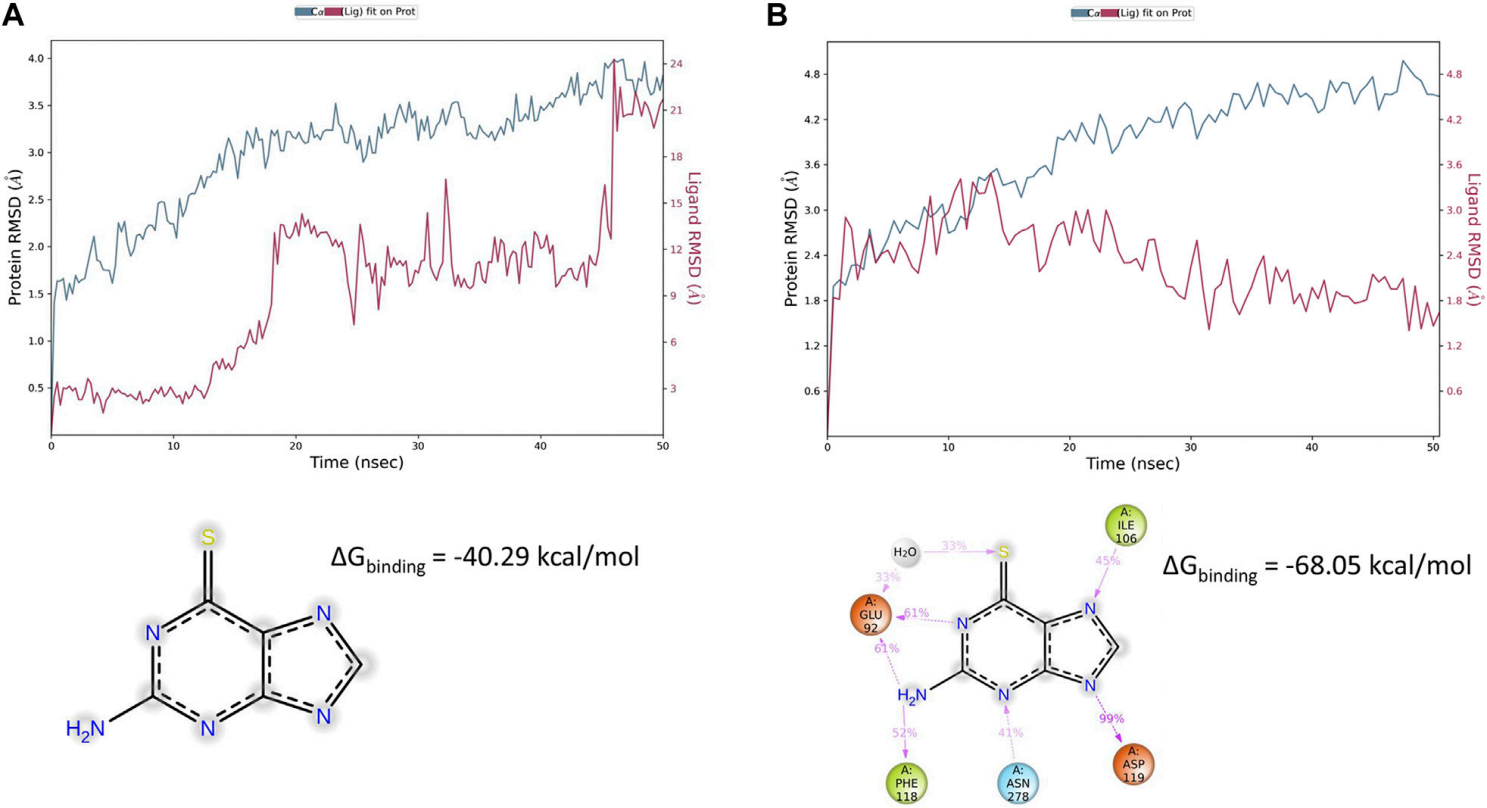
Thioguanine (6TG) interaction with hMC1 and hMC5 receptors during 50 ns simulation. **(A)** hMC1R root mean square fluctuation (blue line) during molecular dynamics simulation of the 6TG/hMC1 complex embedded in an explicit membrane model. 6TG RMSD is represented with a red line. The frequency of thioguanine contacts with hMC1R was below 35% during 50 ns simulation, and 6TG was preferentially exposed to the solvent, outside of the hMC1R-binding pocket. **(B)** hMC5R root mean square fluctuation (blue line) during molecular dynamics simulation of the 6TG/hMC5 complex embedded in an explicit membrane model. 6TG RMSD is represented with a red line. The frequency of thioguanine contacts with hMC5R was above 30% during the 50 ns simulation. Tioguanine was found to bind with a high frequency to Glu92 (also through a water bridge), Ile 106, Asp 119, Phe 118, and Asn 278.

Virtual screening carried out by Gesualdo et al. (2021) provided evidence for 6TG favorable binding for the hMC1 receptor (ΔG_binding_ = −23 kcal/mol), but virtual screening on hMC5R did not evidence any binding for 6TG. We indeed carried out extreme precision molecular docking of 6TG to predict the binding at hMC1 and hMC5; therefore, complexes have been optimized through the MM-GBSA calculation. We found that for the 6TG/hMC5R complex, the binding was characterized by a lower ΔG_binding_ −68.05 kcal/mol, compared to the ΔG_binding_ predicted for the optimized 6TG/hMC1R complex, −40.29 kcal/mol (Figure 2). The difference of approximately 20 kcal/mol for the 6TG/hMC1 receptor complex, as previously reported by Gesualdo et al. (2021), is related to the difference in MM-GBSA rescoring carried out within the virtual screening process, which does not consider the implicit model for the membrane.

### 3.2 Molecular dynamics simulations

**FIGURE 3:**
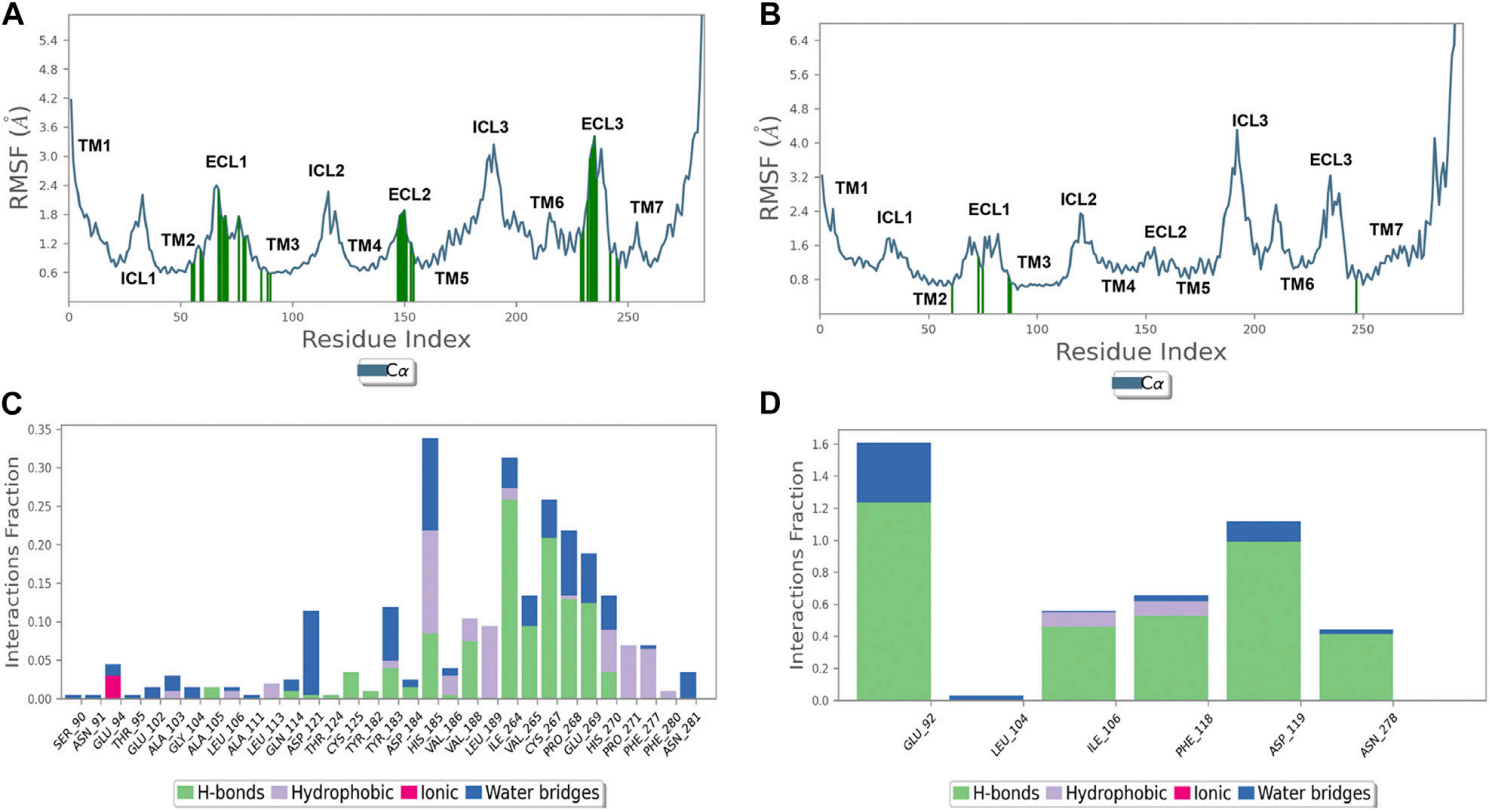
Thioguanine (6TG) binds with the hMC5 receptor with few residues but with a high frequency. **(A)** hMC1R root mean square fluctuation (RMSF, blue line) and 6TG contacts (green lines). **(B)** hMC5R root mean square fluctuation (RMSF, blue line) and 6TG contacts (green lines). **(C)** Frequency (interaction fraction) of contacts of 6TG with hMC1R during 50 ns of simulation. Tioguanine binds to hMC1R through several contacts but with a frequency below 35% (interaction fraction <0.35). **(D)** Frequency (interaction fraction) of contacts of 6TG with hMC1R during 50 ns of simulation. Tioguanine binds to hMC5R with few contacts but with a high frequency, also above 150% (interaction fraction >1.5). Interaction fraction above 1 stands for multiple forms of interaction with a given amino acid (H-bond, water bridge, hydrophobic, and coulombic).

To explain ΔG_binding_ differences between 6TG/hMC1 and 6TG/hMC5 complexes, we carried out 50 ns of molecular dynamics simulation of these two complexes in an explicit water–membrane model. We found that besides a greater number of contacts of 6TG at hMC1R, compared to the 6TG/hMC5 complex, 6TG/hMC1 was characterized by increased protein RMSF (particularly at the first extracellular loop, ECL1) and increased ligand RMSD, in comparison to the 6TG/hMC5 complex (Figures 2, 3).

Additionally, the number of contacts of 6TG at hMC1 had a lower frequency during 50 ns simulation, compared to the frequency of contacts of 6TG at the hMC5 receptor (Figure 3). Overall, the computational approaches evidenced that 6TG binding at the hMC5 receptor is more stable than binding at the hMC1 receptor.

### 3.3 Cell viability in HUVECs

**FIGURE 4:**
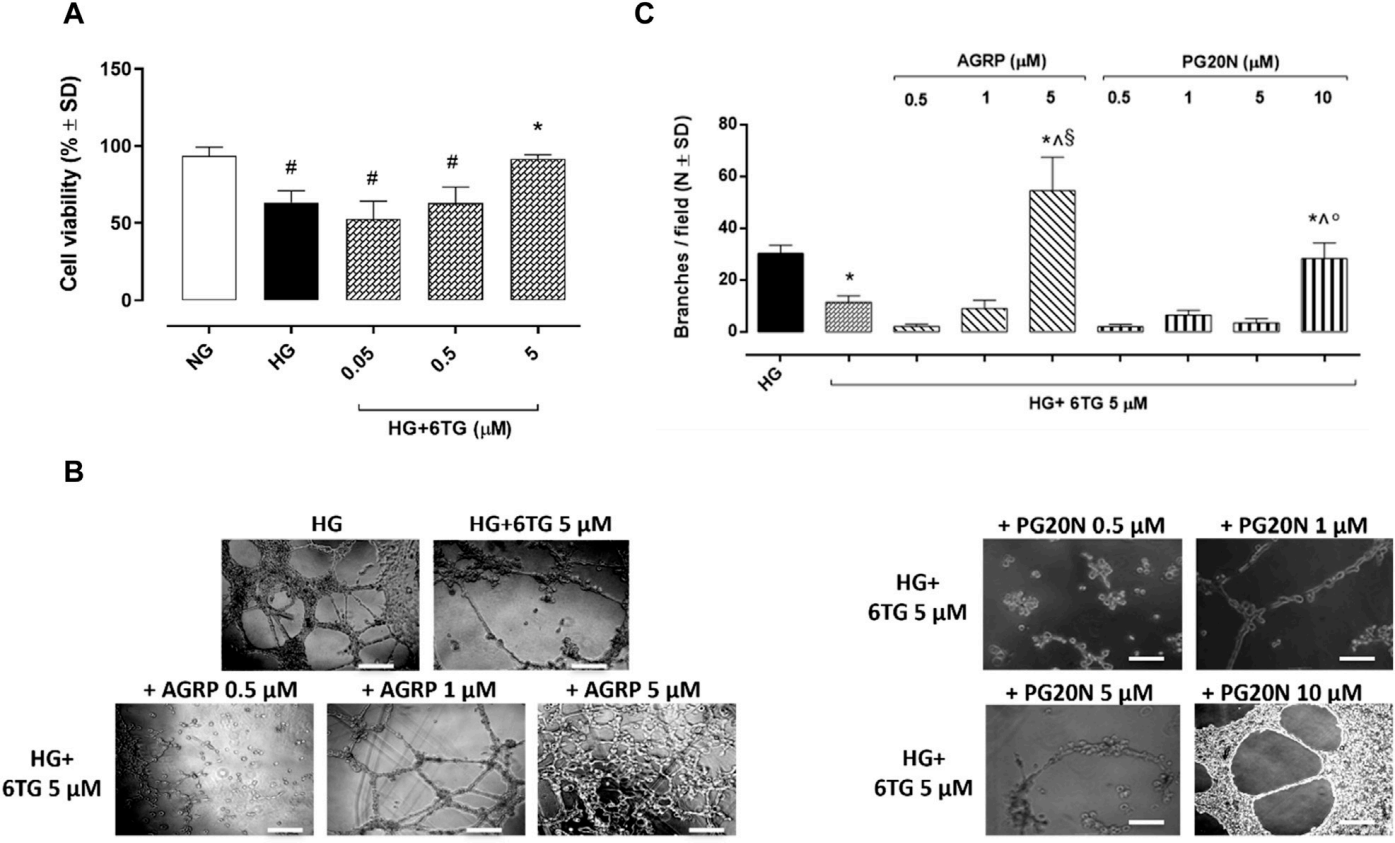
Effects of 6TG on HUVEC viability and angiogenesis under high glucose conditions. **(A)** MTT assay assessing HUVEC viability. Cells were cultured in normal glucose (5 mmol/L) (NG), high glucose (25 mmol/L) (HG), and treated with 6TG 0.05–0.5–5 µM (HG+6TG). Cell viability was reported as a percentage (%) of NG ± SD (*n* = 4). ^#^ *p* < 0.05 *vs.* NG; *p < 0.05 *vs.* HG. **(B)** Representative images of the Matrigel assay for **(C)** the number of branches/field (as a marker of angiogenesis) formed by HUVEC (N = 4) cultured in high glucose (25 mmol/L) (HG) and treated with 6TG 5 µM (HG+6TG) alone and combined with the MCR1 antagonist AGRP (0.5–1–5 µM) (HG+6TG + AGRP) or with the MCR5 antagonist PG20N (0.5–1–5–10 µM) (HG+6TG + PG20N). *p < 0.05 *vs*. HG; ^ *p* < 0.05 *vs.* HG+6TG 5; ^§^ *p* < 0.05 *vs.* HG+6TG + AGRP (0.5–1); °*p* < 0.05 *vs*. HG+6TG + PG20N (0.5–1–5). Scale bar: 100 µm.

6TG (0.05–0.5–5 µM) did not show any toxic effects in HUVECs exposed to normal glucose (5 mmol/L, NG) and has not reduced cell viability (Supplementary Figure S1S). Similarly, cell viability of HUVECs exposed to high glucose (25 mmol/L, HG; 94% ± 5%) did not show any reduction when treated with 6TG 0.05 µM (89% ± 1%, *p* > 0.05 *vs*. HG) and 0.5 µM (89% ± 10%, *p* > 0.05 *vs*. HG), while 6TG 5 µM significantly increased HUVEC viability (120% ± 10%, *p* < 0.05 *vs*. HG) (Figure 4A).

### 3.4 Angiogenesis assessment in HUVECs

The number of branches in HUVECs exposed to HG (30 ± 3) was significantly reduced by 6TG 5 µM (12 ± 2, *p* < 0.05 *vs.* HG). The anti-angiogenic effect of 6TG 5 µM on HUVECs exposed to HG was eradicated by the MC3R antagonist AGRP 5 µM (55 ± 13, *p* < 0.05 *vs.* HG + 6TG 5) and the MC5R antagonist PG20N 10 µM (28 ± 6, *p* < 0.05 *vs*. HG + 6TG 5). 6TG 5 µM co-treatment with AGRP 0.5–1 µM (respectively, 2 ± 1 and 9 ± 3, both *p* > 0.05 *vs*. HG + 6TG 5) or PG20N 0.5–1–5 µM (respectively, 2 ± 1, 7 ± 2 and 4 ± 2, all *p* > 0.05 *vs*. HG + 6TG 5) had no effects on HUVECs exposed to HG (Figures 4B, C).

### 3.5 Fluorescein angiography assessment

**FIGURE 5:**
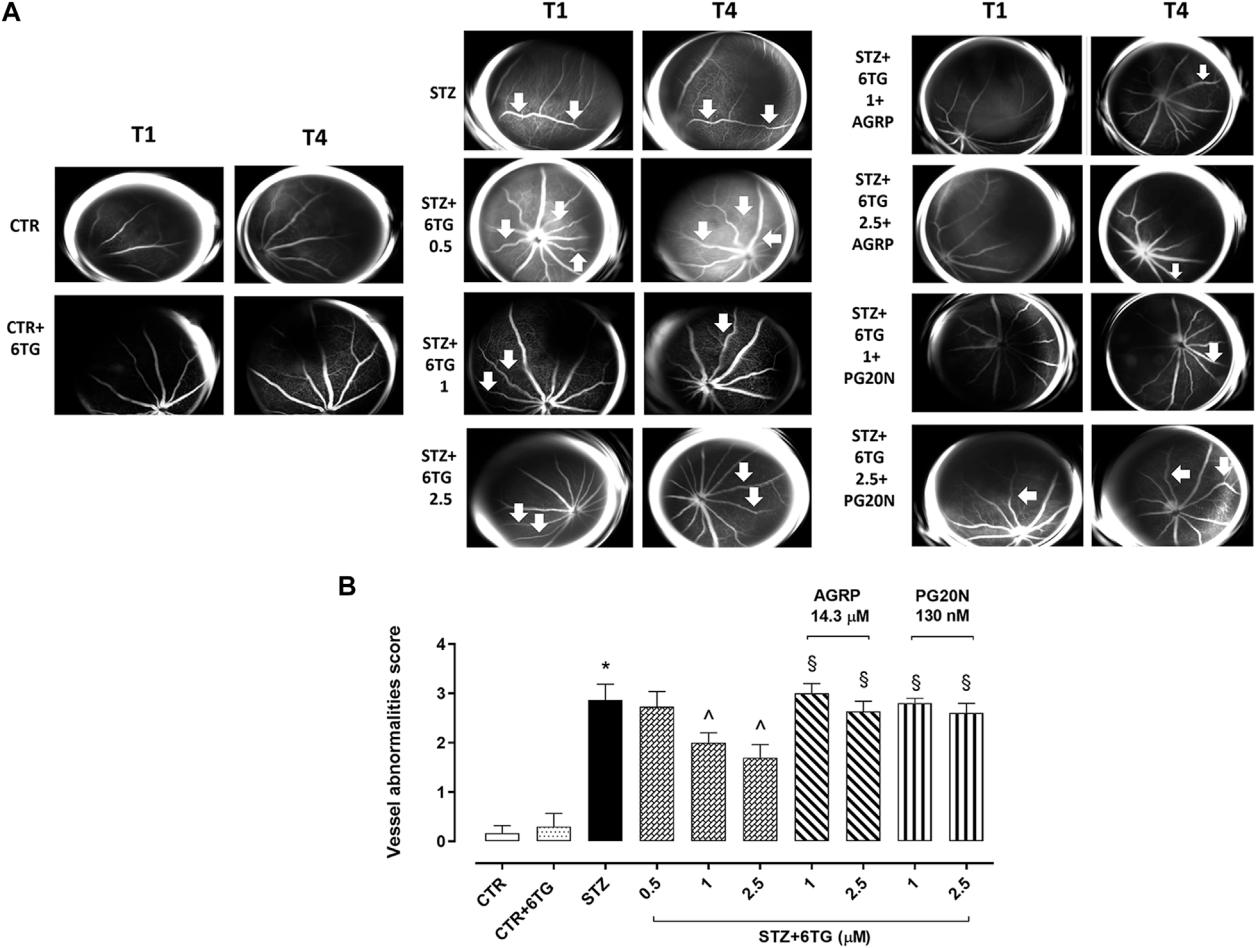
Effects of 6TG on retinal vascular alterations in diabetic mice. **(A)** Representative FA analyses showing the mouse retina (*n* = 4 per group) after 4 weeks of STZ (T1) and after 12 weeks from T0 (T4). CTR: normal vascularization; CTR+6TG normal vessel caliber and course; STZ: irregular vessel caliber and thinning, with marked vessel tortuosity (arrow); STZ+6TG 0.5: microaneurysm and arteriovenous nicking (arrow); STZ+6TG 1: diffused vessel tortuosity (arrow); STZ+6TG 2.5: marked vessel thinning, rare microaneurysms (arrow); STZ+6TG 1 + AGRP: irregular vessel caliber with stacking of red blood cells and blood column stasis (arrow); STZ + 6TG 2.5 + AGRP: irregular vessel caliber and vessel thinning (arrow); STZ + 6TG 1 + PG20N: hyperfluorescent area along the vessel course as microaneurysm (arrow); STZ + 6TG 2.5 + PG20N: irregular vessel caliber and thinning (arrow). **(B)** Vessel abnormalities at T4 graded from 0 to 4, based on the presence of vessel thinning, tortuosity, venous beading, and rosary-like vessels. CTR: non-diabetic control mice; CTR+6TG: non-diabetic mice receiving 6-TG (2.5 mg/kg) intravitreally; STZ: diabetic mice receiving PBS (pH 7.4) intravitreally; STZ+6TG: diabetic mice receiving intravitreal injections of 6-TG (0.5–1–2.5 mg/kg); STZ + 6TG + AGRP: mice receiving intravitreal injections of the MCR1 antagonist AGRP (14.3 µM) and 6-TG (1–2.5 mg/kg); STZ+6TG + PG20N: diabetic mice receiving intravitreal injections of the MCR5 antagonist PG20N (130 nM) and 6-TG (1–2.5 mg/kg). *p < 0.05 *vs*. CTR; ^ *p* < 0.05 *vs*. STZ; ^§^ *p* < 0.05 *vs*. STZ+6TG (same dose).

FA evaluations in non-diabetic mice receiving intravitreal injections of 6TG 2.5 mg/kg (CTR + 6TG group) evidenced a retinal VAs score (0.3 ± 0.2) like non-diabetic mice (CTR group; 0.2 ± 0.1), with a normal vessel caliber and course. At 14 weeks (T4) after STZ injection, retinal VAs were evident in both diabetic mice (STZ group), showing irregular vessel caliber and thinning with marked vessel tortuosity (2.9 ± 0.3, *p* < 0.05 *vs*. CTR), and diabetic mice receiving intravitreal injections of 6TG at the dose of 0.5 mg/kg (STZ + 6TG 0.5 group) showed the presence of microaneurysms and arteriovenous nicking (2.7 ± 0.3, *p* > 0.05 *vs*. STZ). Conversely, higher 6TG doses (1 and 2.5 mg/kg) intravitreally injected in diabetic mice (STZ + 6TG 1 and STZ + 6TG 2.5 groups) significantly reduced the retinal VAs score. In particular, the 1 mg/kg STZ + 6TG group showed diffused vessel tortuosity (2.0 ± 0.2, *p* < 0.05 *vs*. STZ) and 2.5 mg/kg STZ + 6TG evidenced marked vessel thinning and rare microaneurysms (1.7 ± 0.3, *p* < 0.05 *vs*. STZ). Both 6TG doses 1 and 2.5 mg/kg were not effective in reducing the VAs score at T0 when intravitreally administered in combination with the MC1R antagonist AGRP 14.3 µM (STZ + 6TG + AGRP group) or the MC5R antagonist PG20N 130 nM (STZ + 6TG + PG20N group). Indeed, STZ + 6TG 1 + AGRP mice evidenced an irregular vessel caliber with stacking of red blood cells and blood column stasis (3.0 ± 0.2, *p* < 0.05 *vs*. STZ + 6TG 1), as well as the STZ + 6TG 2.5 + AGRP group, showing an irregular vessel caliber and vessel thinning (3.0 ± 0.2, *p* < 0.05 *vs*. STZ + 6TG 2.5). Similarly, in STZ + 6TG 1 +PG20N and STZ + 6TG 2.5 + PG20N groups, a hyperfluorescent area along the vessel course as microaneurysm (2.8 ± 0.1, *p* < 0.05 *vs*. STZ + 6TG 1) and an irregular vessel caliber and thinning were present, respectively (2.6 ± 0.2, *p* < 0.05 *vs.* STZ + 6TG 2.5) (Figures 5A, B).

### 3.6 Retinal CD34 staining in STZ mice

**FIGURE 6:**
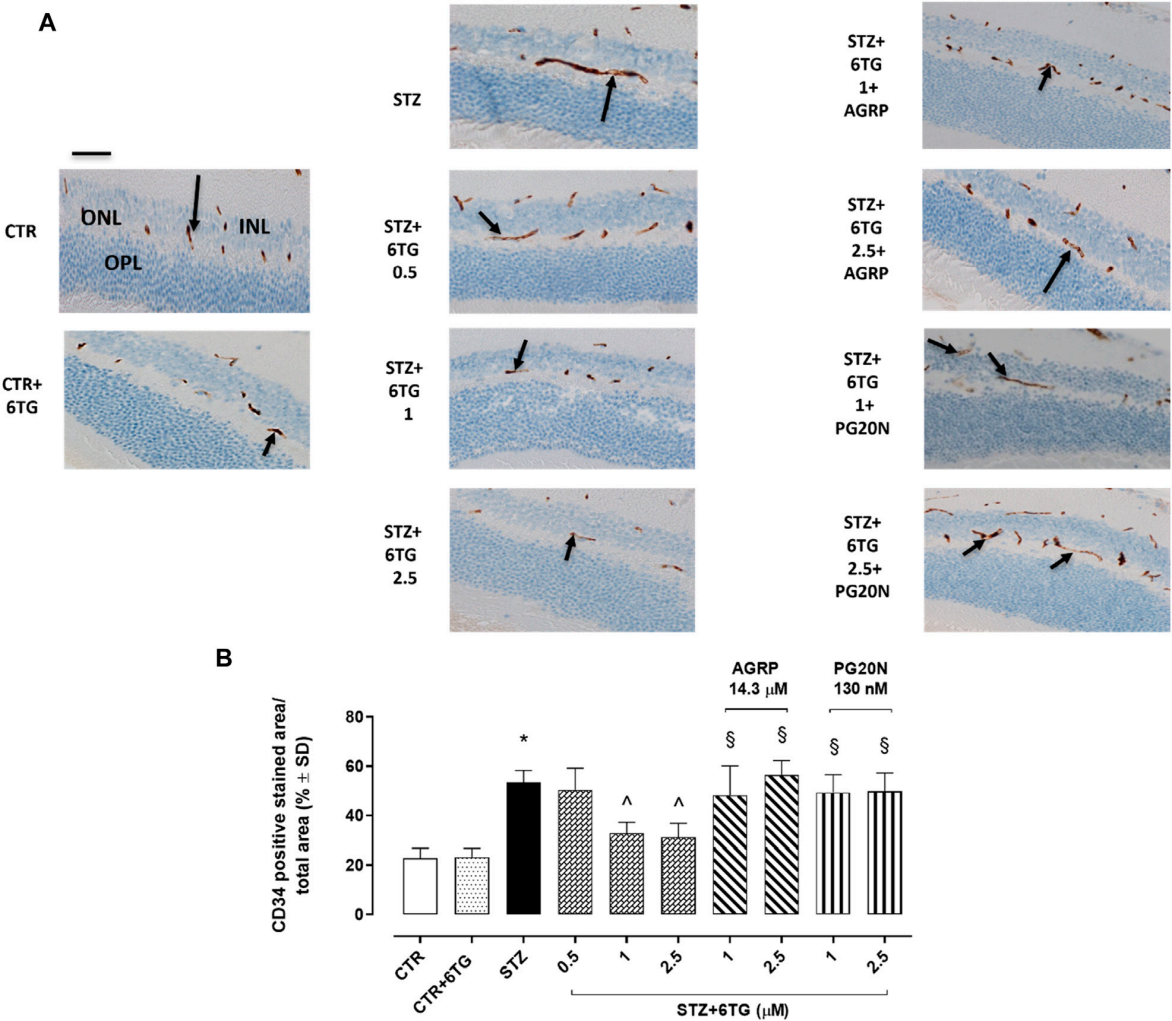
Effects of 6TG on CD34 retinal neo-angiogenesis in diabetic mice. **(A)** Representative retinal IHC images showing C34 labeling (black arrow), as a marker of neo-angiogenesis after 12 weeks from T0 (T4) in CTR, CTR + 6TG, STZ, STZ + 6TG, STZ + 6TG + AGRP, and STZ + 6TG + PG20N groups. Scale bar: 20 µm. **(B)** Percentage (%) of the CD34-positive stained area/total area analyzed (*n* = 4). *p < 0.05 *vs.* CTR; ^ *p* < 0.05 *vs.* STZ; ^§^ *p* < 0.05 *vs.* STZ+6TG (same dose). CTR: non-diabetic control mice; CTR+6TG: non-diabetic mice receiving 6-TG (2.5 mg/kg) intravitreally; STZ: diabetic mice receiving PBS (pH 7.4) intravitreally; STZ+6TG: diabetic mice receiving intravitreal injections of 6-TG (0.5–1–2.5 mg/kg); STZ+6TG + AGRP: mice receiving intravitreal injections of the MCR1 antagonist AGRP (14.3 µM) and 6-TG (1.–2.5 mg/kg); STZ+6TG + PG20N: diabetic mice receiving intravitreal injections of the MCR5 antagonist PG20N (130 nM) and 6-TG (1.–2.5 mg/kg); INL: inner nuclear layer; IPL: inner plexiform layer; ONL: outer nuclear layer; OPL: outer plexiform layer.

CTR and CTR + 6TG groups exhibited weak CD34-positive retinal staining (respectively, 23% ± 4% and 22% ± 3%) as a marker of neo-angiogenesis (Di Filippo et al., 2014), predominantly in the outer plexiform layer (OPL) and in the inner nuclear layer (INL). This was significantly increased in the OPL and INL of STZ mice (53 ± 5, *p* < 0.05 *vs*. HG) and STZ mice receiving 6TG 0.5 mg/kg intravitreally (STZ+6TG 0.5; 50 ± 9, *p* > 0.05). Conversely, intravitreal injections of 6TG 1 mg/kg and 2.5 mg/kg were able to significantly reduce CD34 retinal labeling in both OPL and INL of STZ mice (respectively, 33% ± 4% and 31% ± 6%, both *p* < 0.05 *vs.* STZ). Interestingly, the combination with AGRP 14.3 µM reverted the 6TG (1–2.5 mg/kg) effects, by showing high CD34 retinal staining (respectively, 48% ± 12% and 56% ± 6%, both *p* < 0.05 *vs.* STZ+6TG at the same dose), as well as the combination of 6TG (1–2.5 mg/kg) with PG20N 130 nM (respectively, 49% ± 7% and 50% ± 7%, both *p* < 0.05 *vs.* STZ + 6TG at the same dose) (Figures 6A, B).

## 4 Discussion

Retinal neovascularization, a PDR hallmark, is a multiphasic process that starts with basal membrane degradation by activated retinal endothelial cells (Kusuhara et al., 2018). Then, these cells migrate and proliferate, leading to sprout formation in the stromal space. The formation of vascular loops is then followed by the capillary tube development and new basal membrane deposition. Each phase of this process represents a potential target for the inhibitory action of angiostatic molecules, potentially able to prevent DR complications and improve DR prognosis (Presta et al., 2002; Barber et al., 2011). Retinal endothelial cell activation is mainly triggered by VEGF-A, an endothelial cell-specific mitogen growth factor (Simons et al., 2016; Gui et al., 2020). Along with other angiogenic factors such as fibroblast growth factor (FGF), placental growth factor (PlGF), platelet-derived growth factor (PDGF), and angiopoietin-1/2 (Ang-1/2), VEGF-A is overproduced in retinal endothelial cells, following hyperglycemia, inflammation, hypoxia, advanced glycation end products (AGEs), and oxidative stress (Gui et al., 2020). In addition to VEGF-A, the expression of VEGF receptors 1 and 2 (VEGFR1 and VEGFR2) is induced by AGEs, stimulating, respectively, endothelial cell sprouting and vascular permeability (Wu et al., 2014; Kanda et al., 2017; Gesualdo et al., 2021). Diabetic macular edema (DME) is strongly associated with DR severity. The current gold standard for DME treatment is using intravitreal injections of anti-VEGF agents or steroids (Tomita et al., 2021). In particular, the current therapeutic approaches, mainly targeting VEGF-A, involve using monoclonal antibodies such as ranibizumab, brolucizumab, faricimab, and bevacizumab (off-label), as well as fusion proteins such as aflibercept (Zhao and Singh, 2018).

Recently, we have shown that the reduction in retinal VEGF-A, VEGFR1, VEGFR2, and blood retinal barrier alterations could be obtained in a DR mouse model by the selective activation of melanocortin receptors 1 and 5 (MC1R and MC5R) (Gesualdo et al., 2021; Rossi et al., 2021). MC1R and MC5R agonists also led to a restoration of antioxidant enzymes in primary retinal cells exposed to high glucose, with the consequent reduction in pro-inflammatory markers (Maisto et al., 2017). Interestingly, by a drug repurposing study, we suggested that, in addition to their selective agonists, MC1R and MC5R could have a good affinity also for some Food and Drug Administration (FDA)-approved compounds (Gesualdo et al., 2021). In particular, the sphingosine 1-phosphate receptor agonist fingolimod, approved for relapsing–remitting multiple sclerosis (RR-MS) therapy (Cohen et al., 2010; Kappos et al., 2010), emerged as a potential MCR1 agonist by a structure-based computational approach (Gesualdo et al., 2021). This was confirmed also in a DR mouse model by the selective MCR1 blockade. Although the molecular dynamic and structural simulations were less straightforward for MCR5, the *in vivo* experiments blocking this receptor suggested the interaction between MC5R and fingolimod (Gesualdo et al., 2021). By the same virtual screening approach as evidenced by Gesualdo et al. (2021), 6TG (labeled as L01) has shown a predicted binding free energy −23 kcal/mol for hMC1R. In the same virtual screening campaign, 6TG has not been identified as a ligand for hMC5R. Therefore, we carried out an extreme precision docking protocol of 6TG to predict again the 6TG pose for hMC1 receptor and *de novo* for hMC5R. These two complexes have been rescored and optimized with MM-GBSA calculations and simulated in an explicit water–POPC) membrane environment. MM-GBSA rescoring for 6TG/hMC1R evidenced a more favorable binding free energy of approximately 20 kcal/mol, when compared to the predicted value reported in Gesualdo et al. (2021), and this difference could be related to the parameters regarding the implicit membrane model, which were added in the hereby presented study. MM-GBSA rescoring evidenced a higher affinity of 6TG for hMC5R, when compared to the value predicted for hMC1R. These data have been confirmed by molecular dynamics simulation of the two complexes. 6TG binds with low and stable RMSD to the hMC5R receptor, and ligand–protein contacts have a greater interaction fraction, when compared to the 6TG-hMC1R complex. These findings correlated with the higher concentration of the hMC5R antagonist used to revert anti-angiogenic effects of 6TG in the *in vitro* experiments hereby presented.

Interestingly, 6TG (2-amino 6-mercaptopurine), along with 6-metilmercaptourine (6MP) riboside, has shown anti-angiogenic properties (Presta et al., 1999; 2002). These are both pro-drugs commonly used in the management of cancer, post-transplant immunosuppression, and autoimmune diseases (Petit et al., 2008). After intestinal and hepatic metabolism, 6MP and 6TG are transformed into thioguanine nucleotides, by replacing the endogenous purine guanine during DNA synthesis, causing DNA strand breaks and modulating gene expression (Vora et al., 2019). In this contest, purine analogs can interfere with molecular mechanisms of intracellular signaling and growth factors, including VEGF (Keshet and Ben-Sasson, 1999). In particular, 6TG was able to inhibit neo-angiogenesis in endothelial GM 7373 cells, in the chick embryo chorioallantoic membrane, and in the rabbit cornea (Presta et al., 1999). Accordingly, 6TG has been found effective as a potential anti-angiogenic molecule since it reduced endothelial cell proliferation induced by VEGF or fibroblast growth factor-2 (FGF2), thus inhibiting endothelial cell sprouting (Presta et al., 2002). *In vivo*, 6TG prevented neovascularization stimulated by VEGF, FGF2, or human leukemia cells (LIK) in the chick embryo chorioallantoic membrane (Presta et al., 2002). Based on the above findings, we tested 6TG effects in the retinal angiogenesis process to assess the prevention or delay of the onset and/or progression of DR using the mouse model. Before the *in vivo* study, we evaluated the safety profile of HUVECs. The compound had no detrimental effects on HUVEC viability under normal or high glucose conditions at all the doses tested. This is in line with the previous evidence reporting 6TG as able to promote apoptosis only in cancer cells (Laera et al., 2019; Li et al., 2020). In particular, 5 µM 6TG significantly increased the cell viability of HUVECs exposed to high glucose and also reduced their vasculogenic activity, confirming its anti-angiogenic effects previously reported in endothelial cells or the chick embryo chorioallantoic membrane, as well as in AML patients showing a reduced bone marrow microvessel density when treated with 6TG (Padró et al., 2000; Presta et al., 2002; Dean, 2012). It is worth noting that AML is frequently associated with severe retinal alterations characterized by retinal microvascular involvement, such as choroidal thickness and abnormalities in retinal circulation, known as leukemic retinopathy (Yang et al., 2023). The anti-angiogenic effects of 6TG (1 and 2.5 mg/kg) were also shown in a DR mouse model by FA analysis, evidencing a reduction in retinal vascular alterations present in diabetic mice, such as irregular vessel caliber, vessel tortuosity and thinning, microaneurysm, or arteriovenous nicking. A further confirmation was obtained by immunohistochemical analysis, showing a remarkable reduction in retinal CD34 staining, a marker of pathological retinal neovascularization, in diabetic mice treated with 6TG (Kollias and Ulbig, 2010; Kady et al., 2017). Interestingly, the selective blocking of MC1R and MC5R reversed the effects of 6TG both *in vitro* and *in vivo* settings. In particular, a higher dose of MC5R antagonist was necessary to eradicate the anti-angiogenic effects of 6TG on HUVECs exposed to high glucose, compared to the MC1R antagonist. This corroborates the results of our simulations, showing that 6TG seems to have a higher affinity for MC5R. The MC1R and MC5R selective antagonist suppressed the protective effects of 6TG on vessel abnormalities, as evidenced by FA analysis. Furthermore, CD34 staining was evident in the retina of diabetic mice receiving 6TG co-administered with MC1R and MC5R antagonists. We already demonstrated that MC1 and MC5 receptor agonists inhibit angiogenesis, decreasing the VEGF-A release, while MC1 and MC5 receptor antagonists increased VEGF-A retinal levels (Gesualdo et al., 2021). Tioguanine has been found to decrease VEGF protein levels through ERK pathway inhibition in malignant glioma cells (U87 cells) (Mukhopadhyay et al., 1998). These findings were also confirmed in breast cancer cells (MCF-7) treated with 10 μM 6TG (Gallwitz et al., 2002). In our study, 6TG elicited anti-angiogenic effects *in vitro* and *in vivo* through MC1 and MC5 activation; however, further studies are needed to investigate the modulation on VEGF-A levels in the retina treated with 6TG. In conclusion, the present findings suggest a new repurposing of 6TG to handle DR. Future research should focus also on topical 6TG delivery using innovative and stable ocular formulation.

## Data Availability

The raw data supporting the conclusion of this article will be made available by the authors, without undue reservation.
