# Supplementary material for: Ocular pharmacological and biochemical profiles of 6-thioguanine: a drug repurposing study
Source: Front Pharmacol. 2024 Mar 25;15:1375805. doi: 10.3389/fphar.2024.1375805 (PMC10999531; doi:10.3389/fphar.2024.1375805)
Supplement: Supplementary file 1 [file Image1.pdf]

Supplementary Material

1 Supplementary Figure

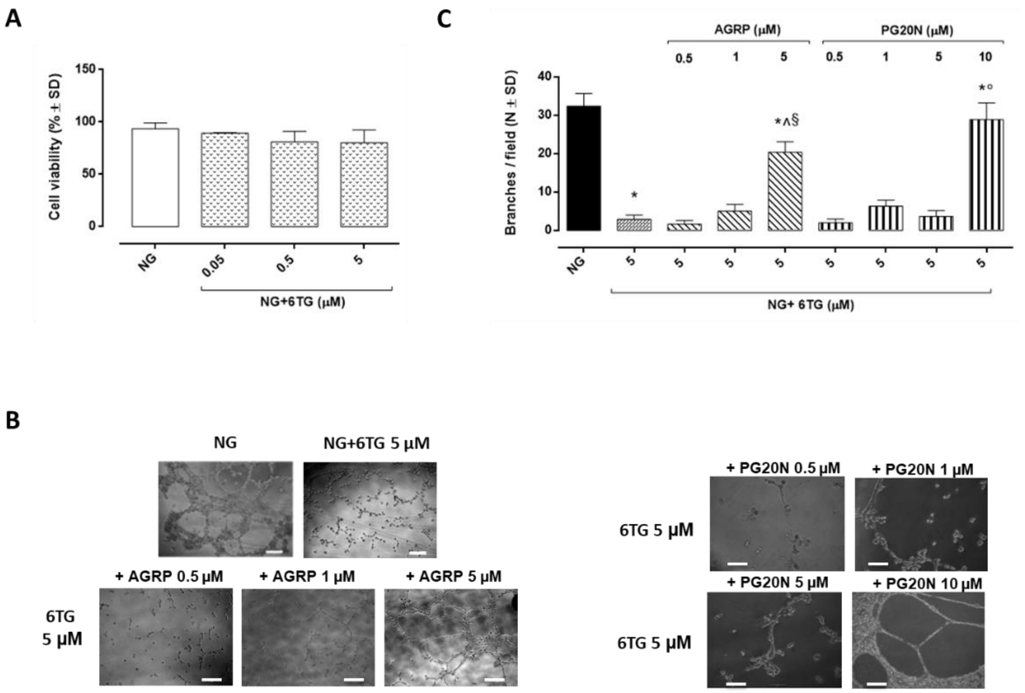

Supplementary Figure 1S. Effects of 6TG on HUVEC viability and angiogenesis in normal glucose. (A) MTT assay assessing HUVECs viability, reported as percentage (%) ± SD (n = 4). Cells were cultured in normal glucose (5 mmol/L) (NG) and treated with 6TG 0.05-0.5-5 μM (NG+6TG). (B) Representative images of Matrigel assay for (C) the number of branches/field (as a marker of angiogenesis) formed by HUVECs cells (n = 4) cultured in normal glucose (5 mmol/L) (NG) and treated with 6TG 5 μM (NG+6TG) alone and combined with MCR1 antagonist AGRP (0.5-1-5 μM) (NG+6TG+AGRP) or with MCR5 antagonist PG20N (0.5-1-5-10 μM) (NG+6TG+PG20N). *P < 0.05 *vs*. NG; ^ P < 0.05 *vs*. NG+6TG 5; § P < 0.05 *vs*. NG+6TG+AGRP (0.5-1); °P < 0.05 *vs*. NG+6TG+PG20N (0.5-1-5). Scale bar: 100 μm.
